# Standard practice in the treatment of unstable pelvic ring injuries: an international survey

**DOI:** 10.1007/s00264-023-05859-x

**Published:** 2023-06-17

**Authors:** Felix Karl-Ludwig Klingebiel, Morgan Hasegawa, Joshua Parry, Zsolt J. Balogh, Ramesh Kumar Sen, Yannik Kalbas, Michel Teuben, Sascha Halvachizadeh, Hans-Christoph Pape, Roman Pfeifer, SICOT Trauma Research Group, Turki Bashir Al-Rouk, Zsolt J. Balogh, Bergita Ganse, Marc Hanschen, Ilir Hasani, Felix Karl-Ludwig Klingebiel, Gleb Korobushkin, Yohei Kumabe, Jeannie McCaul, Joshua A. Parry, Mohamed Rashed, Jordan Saveski, Hemant Sharma, Mohammed Zarti, Roman Pfeifer, Boris A. Zelle

**Affiliations:** 1 Department of Traumatology, University Hospital Zurich, Rämistrasse 100, 8091 Zurich, Switzerland; 2 Department of Surgical Research, Harald Tscherne Laboratory for Orthopaedic and Trauma Research, Zurich University Hospital, Zurich, Switzerland; 3 Division of Orthopaedic Surgery, John A. Burns School of Medicine, University of Hawai’i, Honolulu, HI USA; 4 Department of Orthopaedics, Denver Health Medical Center, University of Colorado School of Medicine, Denver, CO USA; 5 Department of Traumatology, John Hunter Hospital, Hunter Medical Research Institute and University of Newcastle, Newcastle, NSW Australia; 6 Department of Orthopaedics, Max Hospital, Mohali, India

**Keywords:** Pelvic ring injuries, Surgical treatment strategy, Damage control, Emergency strategies

## Abstract

**Purpose:**

Unstable pelvic ring injury can result in a life-threatening situation and lead to long-term disability. Established classification systems, recently emerged resuscitative and treatment options as well as techniques, have facilitated expansion in how these injuries can be studied and managed. This study aims to access practice variation in the management of unstable pelvic injuries around the globe.

**Methods:**

A standardized questionnaire including 15 questions was developed by experts from the SICOT trauma committee (Société Internationale de Chirurgie Orthopédique et de Traumatologie) and then distributed among members. The survey was conducted online for one month in 2022 with 358 trauma surgeons, encompassing responses from 80 countries (experience > 5 years = 79%). Topics in the questionnaire included surgical and interventional treatment strategies, classification, staging/reconstruction procedures, and preoperative imaging. Answer options for treatment strategies were ranked on a 4-point rating scale with following options: (1) always (A), (2) often (O), (3) seldom (S), and (4) never (N). Stratification was performed according to geographic regions (continents).

**Results:**

The Young and Burgess (52%) and Tile/AO (47%) classification systems were commonly used. Preoperative three-dimensional (3D) computed tomography (CT) scans were utilized by 93% of respondents. Rescue screws (RS), C-clamps (CC), angioembolization (AE), and pelvic packing (PP) were observed to be rarely implemented in practice (A + O: RS = 24%, CC = 25%, AE = 21%, PP = 25%). External fixation was the most common method temporized fixation (A + O = 71%). Percutaneous screw fixation was the most common definitive fixation technique (A + O = 57%). In contrast, 3D navigation techniques were rarely utilized (A + O = 15%). Most standards in treatment of unstable pelvic ring injuries are implemented equally across the globe. The greatest differences were observed in augmented techniques to bleeding control, such as angioembolization and REBOA, more commonly used in Europe (both), North America (both), and Oceania (only angioembolization).

**Conclusion:**

The Young-Burgess and Tile/AO classifications are used approximately equally across the world. Initial non-invasive stabilization with binders and temporary external fixation are commonly utilized, while specific haemorrhage control techniques such as pelvic packing and angioembolization are rarely and REBOA almost never considered. The substantial regional differences’ impact on outcomes needs to be further explored.

## Introduction

Unstable pelvic ring injuries represent a challenging injury for trauma surgeons. Established classification systems, imaging, resuscitative methods, implant options, and surgical techniques have allowed for an improved understanding, stabilization, and treatment of these injuries [1–3].

Unstable pelvic ring according to Young and Burgess which is based on the injury mechanism is defined as anterior poster compression (APC) type II/III, lateral compression (LC) type III, vertical shear (VS), and combined mechanism (CM) [4, 5]. Using the AO/Tile classification which relies on vertically and rotational stability, type B (partially unstable) and C (completely unstable) are indicative of an unstable pelvic ring situation [6]. The terminology of an unstable pelvic ring is used due to the concomitant disruption of major ligaments and injuries to the soft tissue and internal organs as well as venous or arterial bleeding in these fracture patterns [7]. This constellation of injuries can result in substantial haemodynamic instability with possible exsanguination and death [8, 9].

Overall, the incidence of operations of the pelvic ring is drastically rising within the last years, whereas especially the usage of minimally invasive procedures with screw fixation seems to have found its way into frequently used practice over the last decade [10]. Nowadays, percutaneous screw stabilization of the posterior pelvic ring is also performed in emergency situation (“antishock/rescue screws”), which provides an alternative to otherwise used techniques such as C-clamp application [11]. In addition, a recent survey among international trauma surgeons provides evidence that percutaneous might provoke a smaller surgical load (second hit phenomenon) in polytraumatized patients [12].

Yet even with potential long-term disability associated with these injuries, no standard treatment algorithm exists, and variability among treating surgeons remains [13]. Still, a recently published treatment algorithm treatment for unstable pelvic ring injuries highlights the relevance of the physiological status on the surgical decision-making and proposes fixation strategies according to the injury pattern [14]. Prior studies examining variability among surgeons have highlighted heterogeneity in most facets of the treatment of these injuries, including timing of definitive fixation, anatomic location of fixation, and treatment of haemodynamically unstable pelvic ring injuries [15–17]. This dearth of high-quality evidence may be the impetus for the substantial management variability associated with these fractures. Yet, within the current literature, there is no investigation into geographical variation of treatment for unstable pelvic ring injuries.

This study aims to evaluate similarities and differences in treatment strategies for unstable pelvic ring care among an international cohort of surgeons. To gain this knowledge, responses were collected from a standardized questionnaire completed by surgeons from an international trauma society representing a large collection of differing nations and geographic regions.

## Materials and methods

### Study design

The initial survey was developed by the SICOT trauma committee and other experts of pelvic ring injuries.

Pilot study: The survey was preliminarily evaluated by experienced trauma surgeons (S.H, M.T R.P, H-C.P) and members of the SICOT trauma committee; annotations and suggestions were implemented.

The survey was then disseminated among members of SICOT, and responses were collected after voluntary participation and submission of survey responses.

### Ethics approval statement

The survey was anonymous and voluntary. All participants agreed to the use of their provided data. The local ethic committee disclosed a general waiver for anonymous surveys.

### Survey

The questionnaire was offered between September 05 and October 05, 2022, consisting of twelve possible selections between four different categories.

*Sociodemographics*: (Gender, country, working experience, level of education, and treatment frequency of unstable pelvic ring injuries); five questions.

*Classification systems*: Options: (1) Denis classification of sacral fractures, (2) Tile/AO classification, (3) Young-Burgess classification; one question.

*Prehospital phase and diagnostic*: Pelvic binders/sheets, preoperative 3D CT; 4-point rating scale: (1) never, (2) seldom, (3) often, (4) always; two questions.

*Initial treatment*: External fixation, rescue screws, C-clamps; 4-point rating scale: (1) never, (2) seldom, (3) often, (4) always; three questions.

*Treatment of bleeding*: Pelvic packing, REBOA, angioembolization; 4-point rating scale: (1) never, (2) seldom, (3) often, (4) always; three questions.

*Secondary surgery and reconstruction*: Staging of surgeries, percutaneous techniques, navigated techniques; 4-point rating scale: (1) never, (2) seldom, (3) often, (4) always; three questions.

The survey function in Google forms (Google LLC, “Standard practice in treatment of unstable pelvic ring injuries”, Accessed 10/05/22, https://forms.gle/ajSqUCktVKaQi7gr8) was used by which anonymity was guaranteed to all participants. The online survey (Appendix 1) was distributed to the members of SICOT. One reminder was sent after four weeks to members of SICOT.

### Statistical analysis

Categorical and ordinal variables are shown as count and percentages and continuous variables as mean, median, or mode (most frequent answer). Groups of continuous variables were compared with Student’s *t*-test. Graphics were created utilizing the R-package ggplot (R Core Team (2019), R Foundation for Statistical Computing, Vienna, Austria (https://www.R-project.org). Statistical analysis was performed using R (R Core Team (2019), R Foundation for Statistical Computing, Vienna, Austria (https://www.R-project.org)) [18]. Stratification by continent was performed. A 4-point rating scale was chosen without a category such as “sometimes” in between “often” and “seldom” to avoid a central tendency, allowing for improved isolation of the actual treatment standards.

## Results

### Participant’s demographics

A total of 358 participants from 80 countries completed the questionnaire. Countries from Asia were most represented (*n* = 177, 50%), followed by African (*n* = 70, 19.8%) and European (*n* = 63, 17.8%) countries. Participants were mostly male (*n* = 331, 92.7%) and most commonly in consultant positions (*n* = 231, 64.5%), as well as head of department (*n* = 48, 13.4%) or fellows (*n* = 46, 12.8%). Experience was stated as > ten years in 48.3% (*n* = 173), 5–10 years in 30.4% (*n* = 109), and < five years in 21.2% (*n* = 76) of responses. The volume of treated unstable pelvic ring injuries was quantified as one to five per months for 66.5% of the participants (*n* = 238), whereas 13.1% (*n* = 47) reported treatment of five to ten unstable pelvic ring injuries per month. Additionally, 8.7% (*n* = 31) of participants stated to treat more than ten unstable pelvic ring injuries per month, and 11.7% (*n* = 42) participating surgeons indicated to not treat any. Summaries and additional demographic information can be seen in Table 1 and Appendix (Table 7).

**Table 1:** Demographics

Demographics	Africa	Asia	Europe	North America	Oceania	South America	Overall
*n*	70	177	63	20	8	16	358
Gender = male (%)	65 (94.2)	169 (95.5)	56 (88.9)	19 (95.0)	6 (75.0)	13 (81.2)	331 (92.7%)
Level of education/training, *n* (%)							
Consultant/attending	44 (62.9)	116 (65.5)	34 (54.0)	16 (80.0)	5 (62.5)	12 (75.0)	231 (64.5%)
Fellow	11 (15.7)	25 (14.1)	8 (12.7)	1 (5.0)	0 (0.0)	1 (6.2)	46 (12.8%)
Head of department	7 (10.0)	22 (12.4)	12 (19.0)	2 (10.0)	3 (37.5)	2 (12.5)	48 (13.4%)
Intern	0 (0.0)	1 (0.6)	1 (1.6)	0 (0.0)	0 (0.0)	0 (0.0)	2 (0.6%)
Resident	8 (11.4)	13 (7.3)	8 (12.7)	1 (5.0)	0 (0.0)	1 (6.2)	31 (8.7%)
Professional experience (years), *n* (%)							
< 5 years	19 (27.1)	39 (22.0)	6 (9.5)	6 (30.0)	2 (25.0)	4 (25.0)	76 (21.2%)
> 10 years	27 (38.6)	77 (43.5)	43 (68.3)	11 (55.0)	4 (50.0)	7 (43.8)	173 (48.3%)
5–10 years	24 (34.3)	61 (34.5)	14 (22.2)	3 (15.0)	2 (25.0)	5 (31.2)	109 (30.4%)
Number of unstable pelvic ring injuries treated in your institution (per month), *n* (%)							
> 10 per month	10 (14.3)	6 (3.4)	10 (15.9)	2 (10.0)	3 (37.5)	0 (0.0)	31 (8.7%)
0 per month	8 (11.4)	25 (14.1)	4 (6.3)	1 (5.0)	0 (0.0)	4 (25.0)	42 (11.7%)
1–5 per month	42 (60.0)	125 (70.6)	39 (61.9)	11 (55.0)	5 (62.5)	12 (75.0)	238 (66.5%)
5–10 per month	10 (14.3)	21 (11.9)	10 (15.9)	6 (30.0)	0 (0.0)	0 (0.0)	47 (13.1%)

### Classification usage

**Fig. 1:**
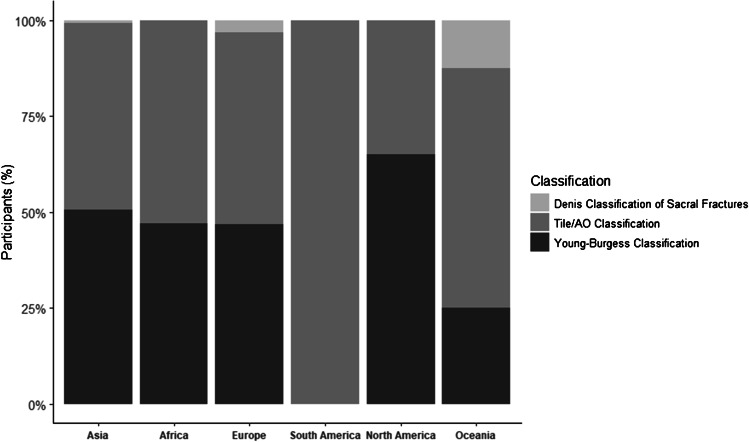
Usage of classification systems across the continents

The Tile/AO classification and the Young-Burgess classification were the most utilized classification systems (Tile/AO = 51.8% (*n* = 183), Y&B = 47% (*n* = 166)). The Denis classification of sacral fractures was only utilized by 1.1% (*n* = 4). Regional analysis indicated South American responders favoured the Tile/AO classification (AO/Tile = 100%, *n* = 16), while North American surgeons showed higher use of the Y&B classification (Y&B = 65%, *n* = 13). Participants from other continents reported a distribution closer to a 50:50 use of AO/Tile and Y&B classifications (Table 2, Fig. 1).

**Table 2:** Usage of classification systems across continents

Classification usage	Africa (*n* = 70)	Asia (*n* = 177)	Europe (*n* = 63)	North America (*n* = 20)	Oceania (*n* = 8)	South America (*n* = 16)	Overall (*n* = 358)
Denis classification of sacral fractures, *n* (%)	0 (0.0)	1 (0.6)	2 (3.2)	0 (0.0)	1 (12.5)	0 (0.0)	4 (1.1)
Tile/AO classification, *n* (%)	37 (52.9)	85 (48.9)	31 (50.0)	7 (35.0)	5 (62.5)	16 (100.0)	183 (51.8)
Young-Burgess classification, *n* (%)	33 (47.1)	88 (50.6)	29 (46.8)	13 (65.0)	2 (25.0)	0 (0.0)	166 (47.0)
Mode	Tile/AO	Y&B	Tile/AO	Y&B	Tile/AO	Tile/AO	Tile/AO

### Pelvic binders

**Fig. 2:**
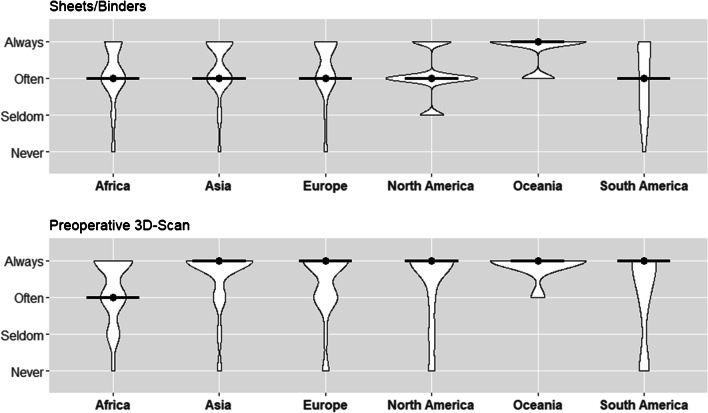
Prehospital management and diagnostics across the continents—usage of pelvic binders (on scene and trauma bay) and preoperative 3D imaging (with median)

The usage of pelvic binders or sheets showed high accordance among all participating surgeons with approximately 87% (*n* = 308) of responders stating they always or often used them in the prehospital setting (median and mode = often). Mode and median of all individual participating surgeons from all continents were either “always” or “often”. Relevant differences in usage were not apparent among continents (Table 3, Fig. 2).

**Table 3:** Prehospital phase and diagnosis performed across continents

Prehospital phase and diagnosis	Africa (*n* = 70)	Asia (*n* = 177)	Europe (*n* = 63)	North America (*n* = 20)	Oceania (*n* = 8)	South America (*n* = 16)	Overall (*n* = 358)
Pelvic binders/sheets usage							
Always, *n* (%)	24 (34.3)	80 (45.5)	29 (46.8)	5 (25.0)	6 (75.0)	6 (40.0)	152 (42.8)
Often, *n* (%)	35 (50.0)	76 (43.2)	26 (41.9)	12 (60.0)	2 (25.0)	4 (26.7)	156 (43.9)
Seldom, *n* (%)	6 (8.6)	14 (8.0)	3 (4.8)	3 (15.0)	0 (0.0)	4 (26.7)	31 (8.7)
Never, *n* (%)	5 (7.1)	6 (3.4)	4 (6.5)	0 (0.0)	0 (0.0)	1 (6.7)	16 (4.5)
Median	Often	Often	Often	Often	Always	Often	Often
Mode	Often	Always	Always	Often	Always	Always	Often
Preoperative 3D computer tomography							
Always, *n* (%)	33 (47.8)	132 (75.4)	36 (57.1)	14 (70.0)	7 (87.5)	9 (56.2)	233 (65.6)
Often, *n* (%)	22 (31.9)	25 (14.3)	19 (30.2)	2 (10.0)	1 (12.5)	3 (18.8)	74 (20.8)
Seldom, *n* (%)	11 (15.9)	9 (5.1)	3 (4.8)	2 (10.0)	0 (0.0)	0 (0.0)	25 (7.0)
Never, *n* (%)	3 (4.3)	9 (5.1)	5 (7.9)	2 (10.0)	0 (0.0)	4 (25.0)	23 (6.5)
Median	Often	Always	Always	Always	Always	Always	Always
Mode	Always	Always	Always	Always	Always	Always	Always

### Preoperative 3D-computed tomography scan (CT scan)

65.5% and 20.8% of participants reported that they always or often used 3D CT scans preoperatively. Within each continent, the mode of responses was “always performing a preoperative 3D CT scans”. This was also observed with mean, with exception of the median from responses of surgeons in Africa, which was found to be often use of preoperative 3D CT scans (median = often) (Table 3, Fig. 2).

### External fixation

**Fig. 3:**
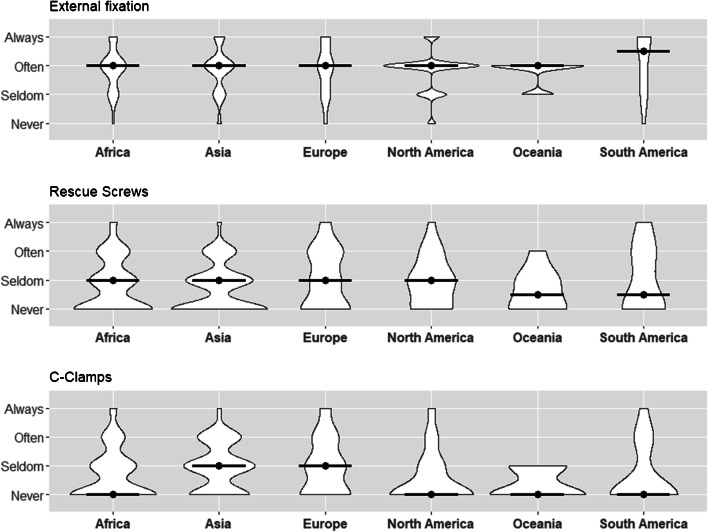
Initial treatment across the continents—usage of emergency fixation devices across the continents (with median)

Among all participants, 51.5% stated often performing external fixation (19.9% = always, 23% = seldom; mode and median = often). No large differences were observed in regard to central tendency. Individuals from all continents stated they often performed external fixation (mode), except for South American respondents, which tended to report always performing external fixation (median = often-always, mode = always) (Table 4, Fig. 3).

**Table 4:** Initial treatment overall continents

Initial treatment	Africa (*n* = 70)	Asia (*n* = 177)	Europe (*n* = 63)	North America (*n* = 20)	Oceania (*n* = 8)	South America (*n* = 16)	Overall (*n* = 358)
External fixation usage							
Always, *n* (%)	12 (17.4)	32 (18.1)	16 (25.4)	2 (10.0)	0 (0.0)	8 (50.0)	71 (19.9)
Often, *n* (%)	38 (55.1)	95 (53.7)	26 (41.3)	13 (65.0)	6 (75.0)	3 (18.8)	184 (51.5)
Seldom, *n* (%)	16 (23.2)	40 (22.6)	16 (25.4)	4 (20.0)	2 (25.0)	4 (25.0)	82 (23.0)
Never, *n* (%)	3 (4.3)	10 (5.6)	5 (7.9)	1 (5.0)	0 (0.0)	1 (6.2)	20 (5.6)
Median	Often	Often	Often	Often	Often	Often-always	Often
Mode	Often	Often	Often	Often	Often	Always	Often
Rescue screw usage							
Always, *n* (%)	3 (4.3)	3 (1.7)	5 (7.9)	1 (5.0)	0 (0.0)	2 (12.5)	15 (4.2)
Often, *n* (%)	14 (20.0)	30 (17.0)	18 (28.6)	4 (20.0)	1 (12.5)	4 (25.0)	71 (19.9)
Seldom, *n* (%)	20 (28.6)	59 (33.5)	15 (23.8)	8 (40.0)	3 (37.5)	2 (12.5)	108 (30.3)
Never,* n* (%)	33 (47.1)	84 (47.7)	25 (39.7)	7 (35.0)	4 (50.0)	8 (50.0)	163 (45.7)
Median	Seldom	Seldom	Seldom	Seldom	Seldom-never	Seldom-never	Seldom
Mode	Never	Never	Never	Seldom	Never	Never	Never
C-Clamp usage							
Always, *n* (%)	3 (4.3)	4 (2.3)	5 (7.9)	1 (5.0)	0 (0.0)	1 (6.2)	14 (3.9)
Often, *n* (%)	10 (14.3)	43 (24.4)	13 (20.6)	2 (10.0)	0 (0.0)	3 (18.8)	71 (19.9)
Seldom, *n* (%)	20 (28.6)	70 (39.8)	20 (31.7)	4 (20.0)	3 (37.5)	1 (6.2)	119 (33.3)
Never, *n* (%)	37 (52.9)	59 (33.5)	25 (39.7)	13 (65.0)	5 (62.5)	11 (68.8)	153 (42.9)
Median	Never	Seldom	Seldom	Never	Never	Never	Never
Mode	Never	Seldom	Never	Never	Never	Never	Never

### Rescue screw (acute percutaneous posterior pelvic ring stabilization) usage

Overall, 45.7% of respondents indicated never utilizing rescue screws and 30.3% reporting seldom use (median = seldom, mode = never). The only continent reaching a mode of “Seldom” was North America, whereas respondents from other continents most commonly answered “Never” using rescue screws, indicating a distinct lack of use outside of North America (Table 4, Fig. 3).

### C-Clamp usage

C-Clamps were reported as rarely used, with 42.9% of all the participating trauma surgeons stated they never used C-clamps, while 33.3% stated seldom use (median and mode = never). The highest rate of use was observed among surgeons from Asia and Europe (median = seldom; mode Asia = seldom, mode Europe = never), whereas participants from other continents most commonly reported never using C-clamps, with calculated median and mode indicating “Never using c-clamps” (Table 4, Fig. 3).

### Pelvic packing

**Fig. 4:**
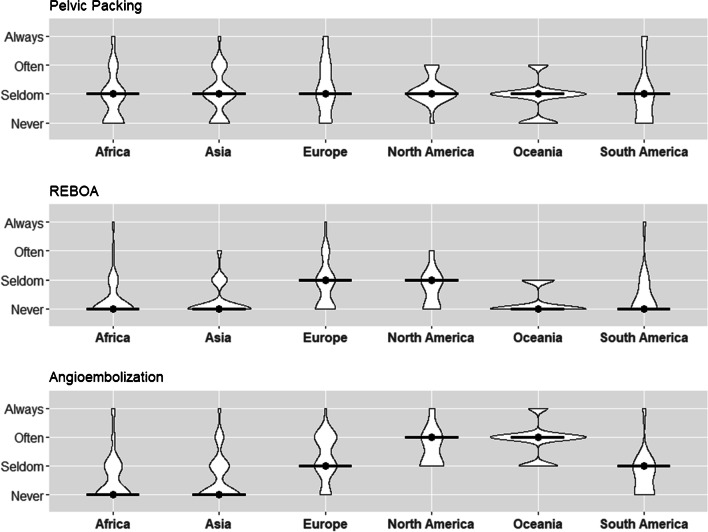
Usage of bleeding control techniques across continents (with median)

Pelvic packing for hemostasis was reported with a central tendency to seldom performed (47%), 28% of participants stated never utilizing pelvic packing, and 19.8% stating often application of the technique. The highest rate of usage was found in surgeons from North America, though 5.3% of respondents still reported never using this technique. There was no difference in central tendencies among all continents (all continents: median and mode = seldom) (Table 5, Fig. 4).

**Table 5:** Treatment of bleeding across continents

Treatment of bleeding	Africa (*n* = 70)	Asia (*n* = 177)	Europe (*n* = 63)	North America (*n* = 20)	Oceania (*n* = 8)	South America (*n* = 16)	Overall (*n* = 358)
Pelvic packing usage							
Always, *n* (%)	4 (5.8)	5 (2.9)	7 (11.1)	0 (0.0)	0 (0.0)	2 (12.5)	18 (5.1)
Often, *n* (%)	11 (15.9)	38 (21.8)	14 (22.2)	4 (21.1)	1 (12.5)	1 (6.2)	70 (19.8)
Seldom, *n* (%)	29 (42.0)	83 (47.7)	26 (41.3)	14 (73.7)	5 (62.5)	7 (43.8)	166 (47.0)
Never, *n* (%)	25 (36.2)	48 (27.6)	16 (25.4)	1 (5.3)	2 (25.0)	6 (37.5)	99 (28.0)
Median	Seldom	Seldom	Seldom	Seldom	Seldom	Seldom	Seldom
Mode	Seldom	Seldom	Seldom	Seldom	Seldom	Seldom	Seldom
REBOA usage							
Always, *n* (%)	2 (2.9)	0 (0.0)	2 (3.2)	0 (0.0)	0 (0.0)	1 (6.2)	5 (1.4)
Often, *n* (%)	2 (2.9)	8 (4.5)	10 (15.9)	2 (10.0)	0 (0.0)	0 (0.0)	22 (6.2)
Seldom, *n* (%)	13 (18.8)	33 (18.8)	25 (39.7)	10 (50.0)	2 (25.0)	4 (25.0)	88 (24.7)
Never, *n* (%)	52 (75.4)	135 (76.7)	26 (41.3)	8 (40.0)	6 (75.0)	11 (68.8)	241 (67.7)
Median	Never	Never	Seldom	Seldom	Never	Never	Never
Mode	Never	Never	Never	Seldom	Never	Never	Never
Angioembolization usage							
Always, *n* (%)	4 (5.8)	5 (2.8)	2 (3.2)	2 (10.5)	1 (12.5)	1 (6.2)	15 (4.2)
Often,* n* (%)	3 (4.3)	21 (11.9)	24 (38.7)	8 (42.1)	5 (62.5)	0 (0.0)	61 (17.2)
Seldom, *n* (%)	21 (30.4)	49 (27.7)	24 (38.7)	9 (47.4)	2 (25.0)	8 (50.0)	115 (32.4)
Never, *n* (%)	41 (59.4)	102 (57.6)	12 (19.4)	0 (0.0)	0 (0.0)	7 (43.8)	164 (46.2)
Median	Never	Never	Seldom	Often	Often	Seldom	Seldom
Mode	Never	Never	Seldom-Often	Seldom	Often	Seldom	Never

### REBOA usage

REBOA use was reportedly rare, with 67.7% of all participants stating no use and 24.7% reporting utilization (median and mode = never). Surgeons from Europe and North America indicated highest proportion of use (Europe: often = 15.9%, seldom = 39.7%; median = seldom, mode = never) (North America: often = 10%, seldom = 50%; median and Mode = seldom), whereas REBOA use by surgeons elsewhere in the world was most commonly reported as rare or never (median and mode = never) (Table 5, Fig. 4).

### Angioembolization

Responses regarding angioembolization indicated rare or infrequent use (seldom = 32.4%, never = 46.2%; median = seldom, mode = never). Surgeons from Africa and Asia indicated least proportion of use (median and mode = never), followed by South America (median and mode = seldom) and Europe (median = seldom, mode = seldom-often). Surgeons from North America and Oceania reported highest usage, with 10.5% always (median = often, mode = seldom) and 42.1% often (12.5% always and 62.5% often; median and mode = often), respectively (Table 5, Fig. 4).

### Percutaneous techniques

**Fig. 5:**
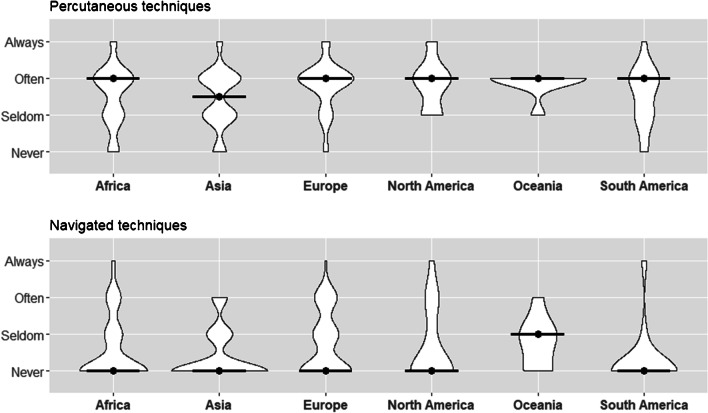
Secondary fixation procedures across the world—usage of percutaneous and navigated techniques (with median)

Percutaneous techniques were reported as often used by 51.1% of respondents (median and mode = often). No substantial differences were observed among surgeons within different continents (median and mode = often; median Asia = seldom-often) (Table 6, Fig. 5).

**Table 6:** Secondary and staged procedures across continents

Secondary surgery and reconstruction	Africa (*n* = 70)	Asia (*n* = 177)	Europe (*n* = 63)	North America (*n* = 20)	Oceania (*n* = 8)	South America (*n* = 16)	Overall (*n* = 358)
Percutaneous techniques usage							
Always, *n* (%)	5 (7.2)	10 (5.7)	6 (9.5)	3 (15.0)	0 (0.0)	1 (6.2)	25 (7.0)
Often, *n* (%)	35 (50.7)	78 (44.3)	42 (66.7)	11 (55.0)	7 (87.5)	8 (50.0)	182 (51.1)
Seldom, *n* (%)	19 (27.5)	64 (36.4)	11 (17.5)	6 (30.0)	1 (12.5)	5 (31.2)	108 (30.3)
Never, *n* (%)	10 (14.5)	24 (13.6)	4 (6.3)	0 (0.0)	0 (0.0)	2 (12.5)	41 (11.5)
Median	Often	Seldom-often	Often	Often	Often	Often	Often
Mode	Often	Often	Often	Often	Often	Often	Often
Navigated techniques usage							
Always, *n* (%)	2 (2.9)	0 (0.0)	1 (1.6)	1 (5.0)	0 (0.0)	1 (6.2)	5 (1.4)
Often,* n* (%)	10 (14.5)	19 (11.0)	14 (22.2)	4 (20.0)	1 (12.5)	0 (0.0)	48 (13.6)
Seldom,* n* (%)	12 (17.4)	33 (19.1)	16 (25.4)	2 (10.0)	4 (50.0)	1 (6.2)	69 (19.5)
Never, *n* (%)	45 (65.2)	121 (69.9)	32 (50.8)	13 (65.0)	3 (37.5)	14 (87.5)	231 (65.4)
Median	Never	Never	Never	Never	Seldom	Never	Never
Mode	Never	Never	Never	Never	Seldom	Never	Never
Secondary/staged procedure							
Always wait for the window of opportunity (5 days), *n* (%)	49 (72.1)	115 (65.3)	21 (33.3)	11 (55.0)	1 (12.5)	13 (81.2)	212 (59.7)
As soon as resuscitation parameters have normalized, *n* (%)	17 (25.0)	41 (23.3)	31 (49.2)	9 (45.0)	6 (75.0)	2 (12.5)	108 (30.4)
Within 72 h, *n* (%)	2 (2.9)	20 (11.4)	11 (17.5)	0 (0.0)	1 (12.5)	1 (6.2)	35 (9.9)
Mode	Window of opportunity	Window of opportunity	Normalized parameters	Window of opportunity	Normalized parameters	Window of opportunity	Window of opportunity

### Navigated percutaneous techniques

Overall, navigated techniques were indicated as rarely utilized, with 65.4% having stated never using navigated techniques, and 19.5% reporting seldom use (median and mode = never). Surgeons from Oceania reported the highest use (median and mode = seldom), though with a small number of surgeons representing the region (*n* = 8) (Table 6, Fig. 5).

### Secondary/staged procedures

**Fig. 6:**
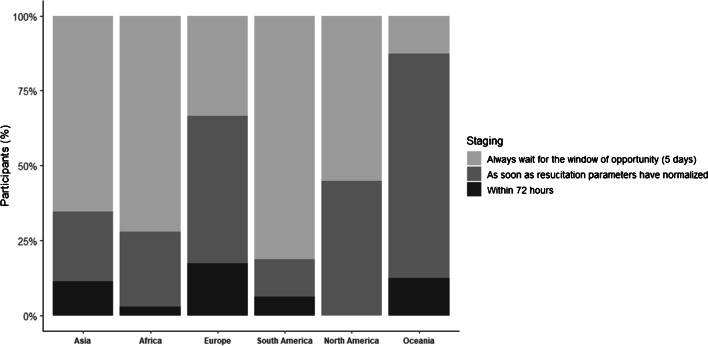
Approaches by which staged/secondary surgery protocols are performed across the continents

Concerning the timing of secondary or staged procedures, 59.7% reported procession of secondary/staged procedures within the window of opportunity, whereas 30.4% reported normalization of resuscitation parameters prior to additional procedures. European (49.2%) and Oceanian (75%) surgeons reported highest proportion of reliance on normalization of resuscitative parameters as dictating their surgical timing. Surgeons from other continents indicated more frequently utilizing the window of opportunity as a gauge for surgical timing. Respondents indicating surgical procedures within 72 h were minimal (9.9%) (Table 6, Fig. 6).

## Discussion

Pelvic ring injuries are associated with high-energy trauma, commonly involving multi-systems, requiring multi-disciplinary treatment and approach [8, 19]. Early approximation or restoration of native pelvic anatomy reduces pelvic volume, facilitating improved intrapelvic clot formation and haemostasis [20, 21].

Even with advancement in imaging and treatment modalities or techniques, along with improved resuscitative methods, broad international agreement in treatment of unstable pelvic ring injuries remains elusive. These fractures represent a challenge for trauma surgeons since they are associated with a high morbidity and mortality due to concomitant haemorrhage, shock, and coagulopathy [22].

Comparison and standardization of different treatment algorithms could potentially lead towards improved outcomes after these debilitating injuries. This study represents one of few studies examining international practice variation around the globe in regard to different facets of treatment for these injuries.

## Study limitations

This expert opinion survey (evidence level IV) is limited to a certain degree. The questionnaire was provided to the entire SICOT society, but the demographic information of the ones not participating could not be retrieved. Therefore, it can only be assumed that the participating cohort is representative for the trauma society. Participants could only choose from and comment to predefined (treatment) options that are most common and determined by the authors. In addition, stratification was performed by continents, which implies homogenous practice in all the underlying countries. There is likely to have huge variability within practice in each continent, which is hidden in this survey. A more detailed (country-wise) analysis would not have been reasonable due to varying sample sizes.

Based on the results of this study:

1. The Young-Burgess and Tile/AO classifications are the most commonly used and in approximate equal proportion.
2. Initial stabilization with binders/sheet is commonly used worldwide.
3. Most surgeons report temporizing stabilization of the pelvic ring with an external fixator.
4. Haemostatic techniques such as pelvic packing and angioembolization are rarely used and REBOA almost never.

Our study participants reportedly use the Young-Burgess and Tile/AO classification systems equally without relevant geographic differences. The Tile/AO classification focuses primarily on posterior pelvic ring stability, whereas the YB classification emphasizes the trauma mechanisms. Prior studies have not established clinically relevant differences when comparing the classification systems in terms of mortality prediction and transfusion/infusion requirements [23]. Still, the interobserver reliability of both classification systems is frequently addressed in literature, whereas it seems that using the Young and Burgess classification provides a better agreement in between surgeons than the AO/Tile classification [24]. This suggests either classification could be used, predicated on consistence of use to improve observer reliability within the institution [24].

In this survey, majority of trauma surgeons, regardless of geographic location, indicated routine prehospital pelvic binder use. Application of a pelvic binder is widely accepted and has been associated with improved haemostasis and mortality reduction in patients with unstable pelvic ring fracture [25–27]. A recent study evaluated the influence of pelvic binder placement accuracy on the resuscitation requirements, whereas displacement of the binder always occurred cranially of the target structure (trochanteric region). No effect on resuscitation parameters or preclinical fluids was seen in this study [28]. Still, correct placement of the binder is of essential importance for the overall outcome [29]. We conclude that the pelvic binder is a simple and relatively benign procedure when applied correctly, with this study’s findings suggesting its use is seemingly uniformly agreed upon.

Overall, the usage of computer tomography seems to increase the agreement, especially in terms of differentiation in between stable and unstable fractures [30]. Additionally, there was wide agreement in preoperative imaging modalities, with most surgeons indicating a 3D CT is obtained preoperatively, further substantiating reports from a recent survey study [17]. The use of 3D CT allows improved evaluation of fracture morphology/classification and characteristics, as well as concomitant injury identification, such as vascular or soft tissue injuries. Prior studies have suggested that obtaining CT scans was associated with better outcome in severely injured patients [31, 32]. Yet, the overall infrastructure and localization of the computer tomography is from essential importance, whereas it needs to be performed quickly especially in haemodynamically unstable patients [33, 34]. The findings in this study are in accordance with the establishment of 3D CT scans as a reliable and beneficial imaging modality, suggesting preoperative 3D CT scans have become a standard diagnostic method for patients with unstable pelvic ring injuries [35].

Unstable pelvic ring injuries often require emergent restoration and mechanical stabilization of pelvic ring anatomy, in part to reduce bleeding and pain in patients [36, 37]. Our findings suggest that the pelvic ring is most commonly temporized with external fixation, with high accordance among surgeons who participated in this study. This is in line with recent literature reporting that surgeons tend to prefer supra-acetabular pin placement over iliac pins in a damage control setting [17, 37].

Temporal fixation with C-clamps was reported to be sparingly used, whereas a recent study reports a beneficial effect of C-clamp application on haemodynamically unstable patients in terms of the hemodynamic stabilization [38], and the high forces imparted with a C-clamp may increase risk of sacroiliac dislocation or over-compression [39]. This troublesome side effect profile has resulted in infrequent use. Gardner first described the usage of acute percutaneous posterior pelvic ring stabilization with “antishock iliosacral screws”, which are also referred to as “rescue screws” [11, 40]. However, this procedure requires substantial surgeon experience; mirrored by our findings, its use is predominated by those in high-volume centres. As such, the seemingly infrequent use of C-clamps and rescue screws might stem from these factors, and others, with unsurprising findings suggesting their use is sparse.

In the setting of persistent haemodynamic instability, further surgical interventions such as pelvic packing and/or angioembolization may be used to control pelvic bleeding [41–44]. Pelvic packing can be quickly performed within 30 min and aims to tamponade venous bleeding, which may also be performed in combination with external fixation [17, 45]. Angioembolization, in general, requires more time and institutional resources [46]. Due to the immense personnel and equipment requirements, not all trauma centres have permanent angioembolization capabilities. It is of note, however, the two techniques are not mutually exclusive, and can be utilized in tandem, and are more likely to be used in high-volume trauma centers [42, 47]. Prior studies have reported an association of angioembolization with reduction of mortality [48]. Yet, it has been discussed that this mortality reduction may be due, in part, to a requisite level of patient stability for patients to be amenable for angioembolization, as there may be increased time requirements for proper equipment and personal to perform the procedure. In contrast, patients who are too unstable to wait for angioembolization may require more expedient methods of hemostasis (e.g., pelvic packing) since angioembolization is usually performed in between two and four h [49]. Additionally, combination pelvic packing (primary) and angioembolization (secondary) has been suggested to be beneficial in patients without haemorrhage controlled primarily [50].

The use of REBOA showed a geographic skew. There was greater use in North America compared to other regions; possibly highlighting its use may require high resource institutions and more robust system processes [51]. In addition, lack of surgeon familiarity with its use, high mortality rates, and frequent complications such as vascular injury, malpositioning, postoperative thrombosis, limb amputation, and tissue necrosis may be reasons for surgeon deterrence of routine use, even in the setting of resource or institutional capabilities [52–55].

The timing of definitive reconstruction in patients with major fractures, such as a pelvic ring fractures, remains contested [56–58]. Early invasive surgery—especially in polytraumatized patients—may exaggerate immunologic and inflammatory responses provoking the “second hit” phenomena, possibly associated with adverse complications and outcomes [59, 60]. In response to this consideration, surgeons may consider physiologic markers or “window of opportunity” when deciding on timing of surgery. A prior study surveying European-based surgeons reported high reliance on a patient'’ physiologic status when determining timing of fracture fixation, rather than the “window of opportunity” when performing a secondary/staged reconstruction [61]. This study congruently aligned with those findings, with most surgeons identifying a patient’s physiology as the main determinant in surgical timing. Yet, even with physiologic status and response of immense importance, a large number of responding surgeons indicated utilization of the “window of opportunity” for timing of fracture fixation. In contrast, some retrospective studies suggest a more dogmatic early definitive stabilization for unstable pelvic ring injuries regardless of the physiology, reporting favorable outcomes [62–64]. Yet, high-quality evidence comparing various methods of determining timing of fracture fixation is lacking.

Recent publications have suggested using multiple parameters to identify when severely injured patient may be stable enough for secondary surgery [65]. Prior studies have incorporated evaluating multiple systems and parameters, such as acid–base balance, body temperature, coagulation, and tissue damage, to aid in improved prediction of early complications when compared to evaluating fewer or single parameters complications [66–68]. Moreover, repeated assessment of physiological parameters in patients with multi-systems injuries allows identification of patients who benefit from early fixation. Intuitively, minimally invasive surgical procedures such as percutaneous pelvic stabilization and navigation techniques allow for early fracture fixation without severely compromising the patient’s physiology commonly associated with open orthopaedic procedures [69–71]. These concepts were exemplified in our study, with percutaneous techniques reported as commonly used.

## Conclusion

This survey of orthopaedic trauma surgeons in regard to treatment of unstable pelvic ring injuries revealed some areas of accordance and discordance in practice. Most surgeons utilize pelvic binders/sheets as an augment to initial stabilization. Additionally, most surgeons report commonly temporizing stabilization of the pelvic ring with an external fixator. Emergency stabilization of the posterior pelvic ring with C-clamps or rescue screws is less commonly performed. Haemostatic techniques such as pelvic packing and angioembolization are infrequently utilized. There was a high degree of variability in regard to determining surgical timing of sequential surgeries, which seems to be a topic under discussion. Most respondents indicated utilizing minimally invasive surgical techniques for definitive fixation of unstable pelvic ring fractures. The current survey study, with international distribution, presents regional agreements and discordances in treatment variation of unstable pelvic ring injuries. Future efforts may include comparison and evaluation of established protocols and treatment algorithms between regions of the world.

## Data Availability

Data, (further) materials and code can be requested individually from the author team. The team of authors reserves the right to evaluate and decide individually to hand out the requested data/materials/code.

## Appendix 1

### Survey Google forms “Standard practice in treatment of unstable pelvis ring injuries”

1. Gender
  - Male
  - Female
2. Level of education/training
  - Intern
  - Resident
  - Fellow
  - Consultant/Attending
  - Head of Department
3. Professional experience (in years)
  -  < 5 years
  - 5–10 years
  -  > 10 years
4. Number of unstable pelvic ring injuries treated in your institution (per month)
  - 0 per month
  - 1–5 per month
  - 5–10 per month
  -  > 10 per month
5. Country of your current employment
  - [Free to fill]
6. What classification system is routinely or primarily used to classify high-energy pelvic ring injuries in your institution?
  - Young-Burgess Classification
  - Tile/AO Classification
  - Denis Classification of Sacral Fractures
7. How often do you use pelvic binders/sheets in pre-hospital/trauma bay setting?
  - Always
  - Often
  - Seldom
  - Never
8. Hof often do you use “Rescue Screws”* for emergency stabilization of unstable pelvic ring fractures? (*Percutaneous screws of the posterior pelvic ring placed in case of emergency)
  - Always
  - Often
  - Seldom
  - Never
9. In your practice, how often in practice is pelvic packing performed in patients with pelvic ring injures and hemorrhage?
  - Always
  - Often
  - Seldom
  - Never
10. In your practice, how often is pelvic external fixation performed in patients with pelvic ring injures and hemorrhage?
  - Always
  - Often
  - Seldom
  - Never
11. How often is REBOA* performed for hemorrhage control in patients with pelvic fractures in your setting? (*Resuscitative endovascular balloon occlusion of the aorta)
  - Always
  - Often
  - Seldom
  - Never
12. How often is angioembolization utilized in patients with pelvic ring injuries and pelvic bleeding?
  - Always
  - Often
  - Seldom
  - Never
13. When do you perform secondary surgery (reconstruction in a staged procedure) in patients with high-energy pelvic ring injures?
  - As soon as resuscitation parameters have normalized
  - Within 72 h
  - Always wait for the window of opportunity (5 days)
14. In your practice, how often is percutaneous techniques with navigation-guided technology used to stabilize pelvic ring injuries?
  - Always
  - Often
  - Seldom
  - Never
15. How often do you obtain a Three-Dimensional (3D) computed tomography (CT) scan to plan treatment of unstable pelvic ring injuries?
  - Always
  - Often
  - Seldom
  - Never
16. Comments (optional)
  - [Free to fill]

## Appendix 2

**Table 7:** List of countries of the participants

Country of your current employment	Participants (n)	Participants (%)
Afghanistan	3	0.8
Algeria	1	0.3
Andorra	1	0.3
Argentina	2	0.6
Australia	7	2
Bahrain	1	0.3
Bangladesh	8	2.3
Belgium	2	0.6
Brazil	3	0.8
Burkina Faso	1	0.3
Cameroon	3	0.8
Canada	3	0.8
Chile	2	0.6
China	1	0.3
Colombia	2	0.6
Cuba	2	0.6
Egypt	20	5.6
England	1	0.3
Ethiopia	2	0.6
France	3	0.8
Germany	3	0.8
Greece	7	2
Haiti	2	0.6
Hungary	2	0.6
India	63	17.8
Indonesia	11	3.1
Iran	1	0.3
Iraq	9	2.5
Ireland	1	0.3
Israel	1	0.3
Japan	4	1.1
Jordan	1	0.3
Kenya	2	0.6
Lebanon	1	0.3
Libya	6	1.7
Luxembourg	1	0.3
Macedonia	1	0.3
Malaysia	5	1.4
Mali	1	0.3
Mexico	3	0.8
Moldova	1	0.3
Morocco	1	0.3
Nepal	17	4.8
New Zealand	1	0.3
Nicaragua	1	0.3
Nigeria	6	1.7
North Macedonia	1	0.3
Oman	2	0.6
Pakistan	9	2.5
Panama	3	0.8
Paraguay	1	0.3
Peru	1	0.3
Philippines	11	3.1
Portugal	3	0.8
Qatar	3	0.8
Romania	2	0.6
Russia	1	0.3
Rwanda	1	0.3
Saudi Arabia	10	2.8
Scotland	1	0.3
Senegal	1	0.3
Somalia	1	0.3
South Africa	6	1.7
Spain	3	0.8
Sri Lanka	1	0.3
Sudan	2	0.6
Switzerland	2	0.6
Syria	1	0.3
Taiwan	1	0.3
Tanzania	5	1.4
Tunisia	2	0.6
Turkey	2	0.6
Uganda	4	1.1
Ukraine	12	3.4
United Arab Emirates	4	1.1
United Kingdom	16	4.5
USA	9	2.5
Venezuela	2	0.6
Yemen	6	1.7
Zambia	5	1.4

## Abbreviations

*REBOA*: Resuscitative endovascular balloon occlusion of the aorta
*Tile/AO*: Tile/AO classification
*Y&B*: Young and Burgess classification
*APC*: Anterior posterior compression
*LC*: Lateral compression
*VS*: Vertical shear
*CM*: Combined mechanism
